# A Survey on the Synthesis of Variolins, Meridianins, and Meriolins—Naturally Occurring Marine (aza)Indole Alkaloids and Their Semisynthetic Derivatives †

**DOI:** 10.3390/molecules28030947

**Published:** 2023-01-18

**Authors:** Marco Kruppa, Thomas J. J. Müller

**Affiliations:** Institut für Organische Chemie und Makromolekulare Chemie, Heinrich-Heine-Universität Düsseldorf, Universitätsstrasse 1, D-40225 Düsseldorf, Germany

**Keywords:** indole alkaloids, marine natural products, natural product syntheses, variolins, meridianins, meriolins

## Abstract

Marine natural products are a source of essential significance due to a plethora of highly diverse biological properties. The naturally occurring (aza)indole alkaloids variolin B (**1**), meridianins (**2**), and their synthetic hybrids meriolins (**3**) exhibit potent kinase inhibitory activities and have aroused considerable interest in the past two decades. Therefore, the immense demand for versatile synthetic accesses to these structures has considerably increased. This review surveys the synthetic pathways to these naturally occurring alkaloids and their semisynthetic derivatives.

## 1. Introduction

Marine ecosystems are an enormous source of chemical diversity. The biological environment is very versatile, and the flora and fauna are exposed to enormous environmental pressure, not only including changes in pressure, salinity, or temperature [1], but also in competition for space, deterrence of predation, and species survival [2]. This led to the development of secondary metabolites in a unique composition, with exceptional frameworks and complex substitution such as halogenation, which rarely occurs in land-based ecosystems [3]. Marine alkaloids, especially indole alkaloids, are of special interest and are produced by a variety of marine sources such as sponges, tunicates, red alga, acorn worms, and symbiotic bacteria [4]. Most isolated alkaloids are biologically active and represent relevant lead structures for pharmacological and medicinal research. The biological properties are diverse with significant antibacterial, antifungal, antiviral, antiparasitic, antitumor, anti-inflammatory, antioxidant, opioid receptor agonistic, antiplasmodial, glucose uptake stimulatory, or immunomodulatory activities [1,5,6]. 

There is no doubt that researchers are extremely captivated by having access to active molecules. Since the amount of naturally produced alkaloids is low, the focus is on the synthesis of (marine) natural products and analogs for biological screenings and potential applications.

**Figure 1:**
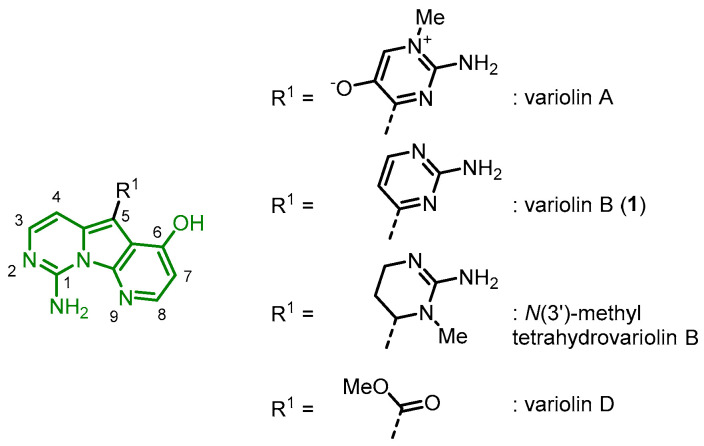
Variolins isolated from the Antarctic sponge *Kirkpatrickia varialosa* [7,8].

Variolins are a class of compounds isolated from the Antarctic sponge *Kirkpatrickia varialosa*. In 1994, Blunt, Munro and coworkers reported the isolation and structural elucidation of four pyridopyrrolopyrimidine alkaloids, variolins A, B, D, and *N*(3′)-methyl tetrahydrovariolin B (Figure 1) [7,8].

Variolins exhibit biological activity, while variolin B (**1**) was the most active compound. It demonstrated in vitro antitumor activity against the P388 murine leukemia cell line and antiviral activity against *Herpes simplex* Type I and *Polio* Type I viruses, whereas variolin A demonstrated weak in vitro activity against the P388 cell line. *N*(3′)-methyl tetrahydrovariolin B was found to be antifungal against *Saccharomyces cerevisiae* and cytotoxic against the HCT 116 human colon cancer cell line. Variolin D, however, did not show any activity, indicating that the 2-aminopyrimidine substituent is necessary for the activity [7,8]. 

**Figure 2:**
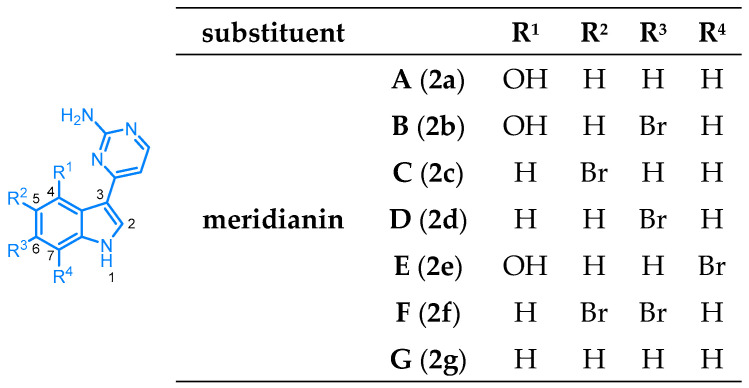
Meridianins **2** isolated from the tunicate *Aplidium meridianum* [9,10].

Different representatives of marine natural products are the indole alkaloids meridianins A to G, isolated from the tunicate *Aplidium meridianum* near the South Georgia Islands. Palermo and coworkers first described the isolation of meridianins A to E in 1998 and later published the identification of two further alkaloids, meridianin F and G [9,10]. Meridianins are brominated indole alkaloids (except for unsubstituted meridianin G) with a 2-aminopyrimidine substituent in 3-position on the indole core (Figure 2). 

Meridianins **2** are structurally related to the variolin family. Not surprisingly, meridianins demonstrate a plethora of biological activities. They display cytotoxic behavior against several cell lines such as LMM3 (murine mammary adenocarcinoma) [9], PTP (foreskin fibroblast cell line), Hep2 (larynx carcinoma), HT29 (colon carcinoma), RD (rabdomyocarcinoma), U937 (myeloid leukemia) [11], vero (monkey kidney fibroblasts), HEPG2 (human hepatoma cells), or LLC-PK1 (pig kidney epithelial cells) [12]. Meridianins appeared to be potent inhibitors of various kinases, with some derivatives inhibiting cyclin-dependent kinases (CDKs) specifically CDK1/cyclin B and CDK5/p25, as well as protein kinases GSK3-β (glycogen synthase kinase-3β isoform), PKA, PKG, and CK1 in a low micromolar range, as well as cAMP- and cGMP-dependent protein kinases. Some derivatives prevent cell proliferation and induce apoptosis [11]. The derivatives with bromine substituents (meridianins B to F (**2b**–**f**)) tend to be more active, with meridianin E (**2e**), having a bromine substituent in the 7-position, being the most active compound. Moreover, comparing meridianin B (**2b**) and D (**2d**), as well as meridianin A (**2a**) and G (**2g**), a hydroxy group in the 4-position, provides a better inhibitory activity [11]. Meridianin C (**2c**) and G (**2g**) showed antiplasmodial activity against *Plasmodium falciparum*, antiparasitic activity against *Leishmania donovani*, and antifungal activity against *Cryptococcus neoformans* [12], while some synthetic analogs display an even more diverse spectrum of biological properties, such as antimalarial and antitubercular [12,13]. 

**Figure 3:**
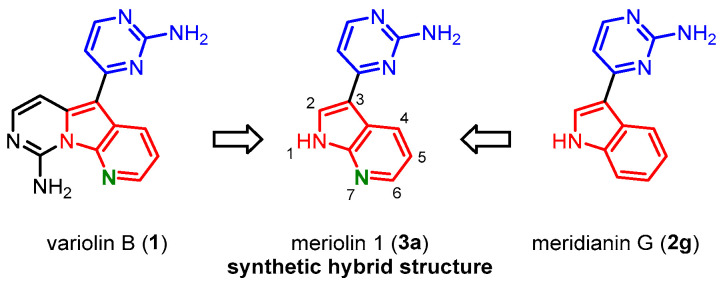
Meriolin 1 (**3a**) as a synthetic hybrid structure of variolin B (**1**) and meridianins **2 [14]**.

Since variolins and meridianins share structural similarities, concerning the 2-aminopyrimidine substituent and the (aza)indole core, as well as their ability to inhibit protein kinases, it was evaluated that a synthetic hybrid structure of variolin B (**1**) and meridianin G (**2g**) would give an interesting lead structure for further biological explorations. The structures of this new compound class were named meriolins **3** (Figure 3). 

Meriolin 1 (**3a**) has first been synthesized by Fresneda und Molina [15], while the name meriolin was shaped by Meijer and co-workers [14,16,17]. The 2-aminopyrimidine-substituted 7-azaindoles exhibit pronounced cytotoxicity against several selected cancer cell lines with IC_50_ values in the nanomolar range [18,19,20] and initiate cell death hallmarks such as cell cycle arrest and decrease in the mitochondrial membrane potential, ΔΨm. Meriolins activate intrinsic apoptotic pathways as well as nonapoptotic cascades leading to necrosis [17,18,21]. Just like the parental natural products, meriolins potently inhibit a broad range of CDKs and appear to be even more active than variolins and meridianins in vitro and in vivo [16,17,18,19,20,22,23,24,25,26].

Due to this versatile range of biological properties and potential pharmaceutical applications, both variolin B (**1**) and meridianin E (**2e**) as well as meriolin **3** derivatives have been submitted to preclinical studies. The CDK-inhibiting active compounds are investigated for the treatment of different types of cancer, including colon cancer or murine and myeloid leukemia [27].

For a detailed description of the biological properties, relevant review articles can be found in the literature [3,22,28]. There are overviews of some syntheses of meridianins and analogs [29,30,31] or variolins [32]. Just recently, Xiao published a brief survey on meridianin syntheses and their biological properties [33]. In 2009, Morris and coworkers summarized syntheses of variolins, meridianins, and meriolins and their biological properties in a review article [34]. Presently, over ten years later, we present an updated and detailed, and, to the best of our knowledge, comprehensive collection of the syntheses of the natural products **1** and **2**, as well as the semisynthetic compounds **3**. For the literature search, Google Scholar, SciFinder, Reaxys, and Web of Science have been used. 

## 2. Synthesis

### 2.1. Syntheses of Variolins

With their unique pyridopyrrolopyrimidine core and pronounced biological activity, variolins, especially variolin B (**1**), were both engaging and challenging for synthetic chemists to provide access to this compound class for biological investigations. Prework has been conducted by Alvarez and coworkers [35,36,37], as well as Vaquero [38,39], Molina and Fresneda [40], and Morris and Anderson [41], who published syntheses towards the pyridopyrrolopyrimidine core. To date, the biosynthesis of variolins is not elucidated. 

#### 2.1.1. First Total Synthesis by Morris and Anderson

**Figure 4:**
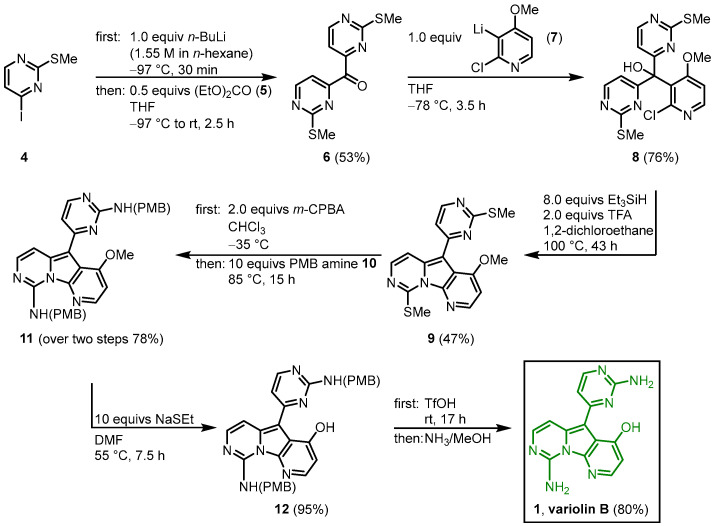
First total synthesis of variolin B (**1**) by Morris and Anderson [42].

The first total synthesis of variolin B (**1**) was achieved by Morris and Anderson in 2001 [42]. Later in 2005, they published the full details of their synthetic strategy together with the synthesis of the synthetic analog desoxyvariolin B [43]. They recognized the C2-symmetry of intermediate **8,** which is cyclized to the pyridopyrrolopyrimidine in the following key step. After halogen lithium exchange in the methylthiopyrimidine **4**, the reaction with diethyl carbonate (**5**) gave the symmetric ketone **6**. The reaction with the lithiated pyridine **7**, followed by the key step tandem deoxygenation and cyclization in the presence of triethylsilane and TFA led to the variolin core structure **9**. The introduction of the amino groups was achieved by oxidizing the dimethylthiol **9** with *m*-chloroperbenzoic acid (mCPBA) to the corresponding disulfoxide, which was reacted with *p*-methoybenzylamine (PMB amine) (**10**) to give the bisprotected amine **11**. Demethylation of **11** and removal of the PMB protecting groups gave the trifluoroacetate salt of the title compound, which was neutralized with concentrated ammonia to give variolin B (**1**) in an eight-step synthesis and an overall yield of 11% (Figure 4) [42].

#### 2.1.2. Synthesis by Molina and Fresneda

**Figure 5:**
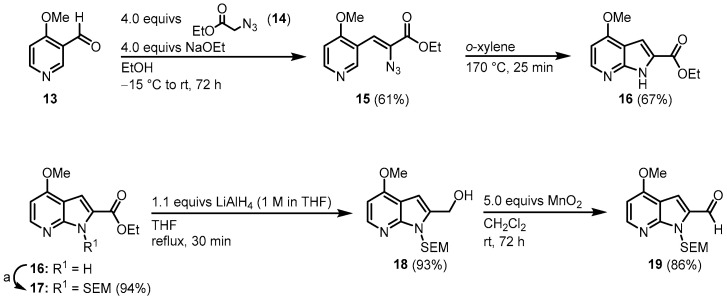
Synthesis of key intermediate azaindole **19**. Reaction conditions for a: first: 1.4 equivs NaH, DMF, rt, 45 min. Then: 1.4 equivs SEM-Cl, rt, 12 h [44].

The next synthetic approach was conducted by Molina and Fresneda, who published their syntheses of **1** in 2002 [44] and a modified synthetic route together with the synthesis of an analog in 2003 [45]. This approach starts with the synthesis of the 7-azaindole **16**. Aldehyde **13** was condensed with azidoacetate **14** and the resulting vinyl azide **15** cyclized to azaindole **16** via a nitrene insertion. After *N*-protection with 2-(trimethylsilyl)ethoxymethyl (SEM), the chloride key intermediate **19** was synthesized in a two-step procedure (Figure 5).

**Figure 6:**
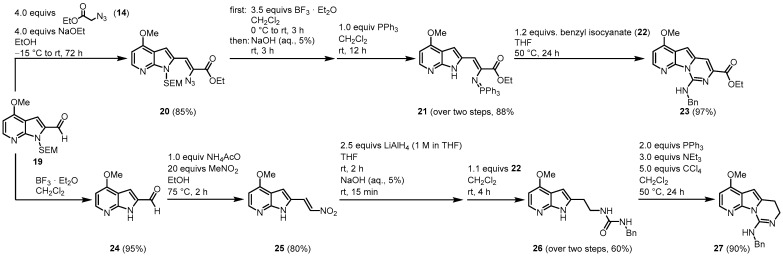
Two approaches to the synthesis of the tricyclic pyridopyrrolopyrimidine structures, **23** and **27** [44].

Next, two different approaches are reported (Figure 6). Aldehyde **19** was similarly condensed as aldehyde **13** to give vinyl azide **20**. After *N*-SEM-deprotection, a Staudinger reaction with triphenylphosphane led to iminophosphorane **21** in a one-pot reaction. Reaction with benzyl isocyanate (**22**) in the key aza-Wittig reaction gave a non-isolable carbodiimide that subsequently cyclized to the desired pyridopyrrolopyrimidine moiety **23**. The cyclization is highly regioselective. In contrast to β-(indol-2-yl)vinyl heterocumulenes, in the present reaction step, no electrocyclic ring closure to the γ-carboline occurred. Instead, the heterocycle is formed by a nucleophilic attack of the amino group on the central carbon atom of the heterocumulene.

**Figure 7:**
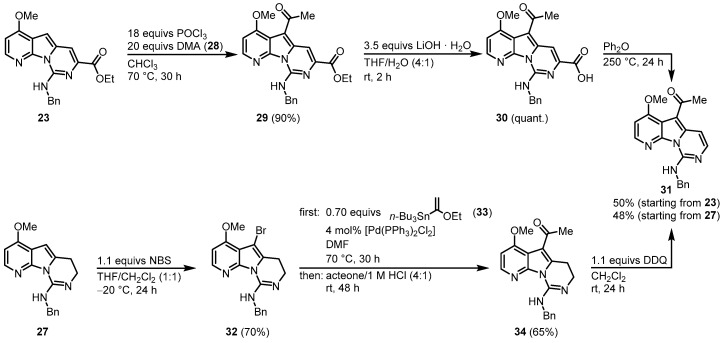
Introduction of an acetyl group at C-5 [44].

Molina and Fresneda developed a second approach to obtain the tricyclic variolin core without the ester group at C-7. After the *N*-SEM-deprotection of **19**, a nitroaldol condensation with nitromethane led to the formation of **25**. Treatment with lithium aluminum hydride gave the corresponding 2-(2-aminoethyl)-7-azaindole, which was sequentially converted to the urea derivative **26** with benzyl isocyanide (**22**) without isolation. The **26** was dehydrated to the carbodiimide, which subsequently cyclized to the dihydropyrimidine **27** using the Appel reagent (CCl_4_/PPh_3_/NEt_3_). Applying both synthetic approaches, an oxygen substituent is placed at C-4 and a nitrogen substituent at C-9. The next step was to introduce the 2-aminopyrimidine ring at C-5, consequently leading both approaches to the acylated intermediate **31**. The reaction of **23** with phosphorus oxychloride and *N*,*N*-dimethylacetamide (DMA) (**28**) allowed the direct introduction of an acetyl group at C-5. Ester hydrolysis led to the carbonic acid **30,** and the thermal treatment forced the formation of intermediate **31** by decarboxylation. The route starting from **27** began with the introduction of a bromine substituent at C-5 and the reaction of bromine **32** with *n*-tributyltin(1-ethoxyvinyl)stannane (**33**) in the presence of dichlorobis (triphenylphosphine)-palladium(II) introduced to the acetyl group at C-5. Oxidation with 2,3-dichloro-5,6-dicyano-1,4-benzoquinone (DDQ) gave the intermediate **31** (Figure 7).

**Figure 8:**
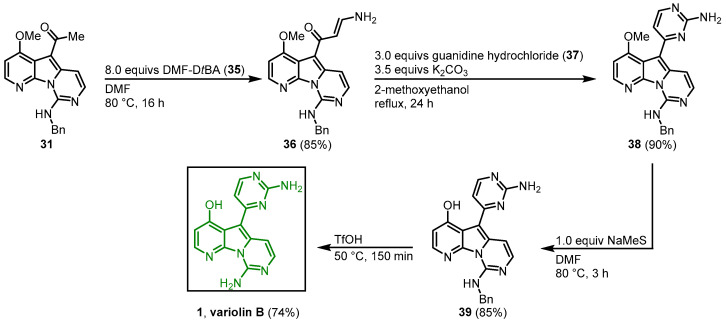
Synthesis of the 2-aminopyrimidine ring to give access to variolin B (**1**) [44].

The 2-aminopyrimidine substituent was synthesized using a protocol developed by Bredereck (Figure 8) [46]. Enaminone **36** was synthesized from **31** with *N*,*N*’-dimethylformamide di-*tert*-butylacetal (**35**) in DMF. Condensation with guanidine hydrochloride (**37**) led to ring closure and formed the desired 2-aminopyrimidine **38**. After demethylation of the hydroxy group at C-4 and the amino group at C-9, variolin B (**1**) was obtained in a 13-step synthesis and an overall yield of 7% [44,45]. 

#### 2.1.3. Variolin B Approach by Alvarez

**Figure 9:**
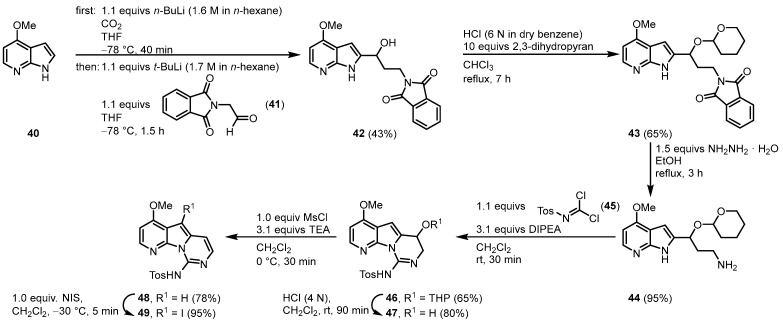
Synthesis of the key intermediate iodide **49** [47].

In 2003, Alvarez published the synthesis of variolin B (**1**) and the synthetic analog desoxyvariolin B [47,48,49]. Starting from 4-methoxy-7-azaindole (**40**), a lithium carboxylate was used as an *N*-protecting group as well as an *ortho*-directing substituent to form a 2-lithio-7-azaindole with a protocol by Katritzky [50]. Reaction with 2-(1,3-dioxoisindolin-2-yl)acetaldehyde (**41**) gave the alcohol **42** that was protected with dihydropyran. *N*-deprotection of **43** by hydrazinolysis gave the aminoacetal **44**. Ring closure was achieved by the reaction with *N*-tosylcarbonimidic dichloride (**45**) and diisopropylethylamine (DIPEA) giving **46** in a diasteriometric mixture in a ratio of 1:1. Removal of the *O*-tetrahydropyran (THP) protecting group and elimination of the resulting hydroxy group by the formation of its mesylate and treatment with triethylamine afforded the pyridopyrrolopyrimidine scaffold (**48**). Regioselective iodination with *N*-iodosuccinimide (NIS) gave the key intermediate **49** (Figure 9).

**Figure 10:**
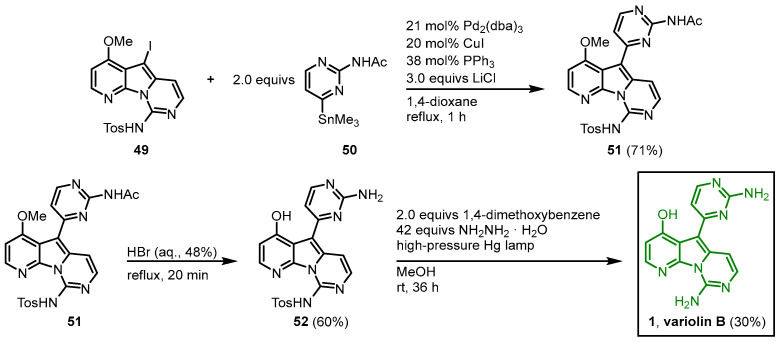
Synthesis of variolin B (**1**) via Stille coupling as a key reaction step [47].

A Stille reaction of **49** and 2-acetylamino-4-trimethylstannylpyrimidine (**50**) in the presence of tris(dibenzylideneacetone)dipalladium(0) afforded **51**. The *O*-demethylation and *N*-acetyl-deprotection were achieved by the treatment of **51** with hydrobromic acid, and after reductive photolysis with hydrazine as a reducing agent and 1,4-dimethoxybenzene as an electron source, the tosyl group was cleaved to give variolin B (**1**) in a 10-step synthesis with an overall yield of 1% (Figure 10).

#### 2.1.4. Synthesis of Variolin B by Burgos and Vaquero

**Figure 11:**
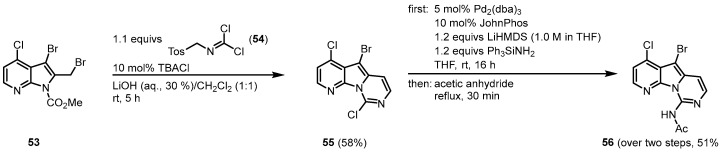
Synthesis of the trihalo core and introduction of the C-9 amino substituent [51].

The 2008 approach by Burgos and Vaquero to synthesize variolin B (**1**) followed the strategy to design the highly functionalized trihalo-substituted pyridopyrrolopyrimidine core **55** and introduce the substituents via palladium-mediated cross-coupling reactions [51,52]. The functionalized 7-azaindole **53** was synthesized from 7-azaindole in six single steps [39]. The **53** was reacted with *N*-tosylmethyl dichloroformimide (**54**) under phase-transfer conditions in the two-phase system LiOH (aq., 30%)/CH_2_Cl_2_ (1:1) with tetrabutylammonium chloride to give the trihalo-substituted compound **55**. The C-9 amino substituent was introduced by a palladium-mediated C-N bond formation, using lithium bis(trimethylsilyl)amide (LiHMDS) and triphenylsilylamine as an ammonia source. The reaction required the use of the ligand [1,1′-biphenyl]-2-yldi-*tert*-butylphosphane (JohnPhos). After *N*-acetyl-protection, **56** was obtained (Figure 11). 

**Figure 12:**
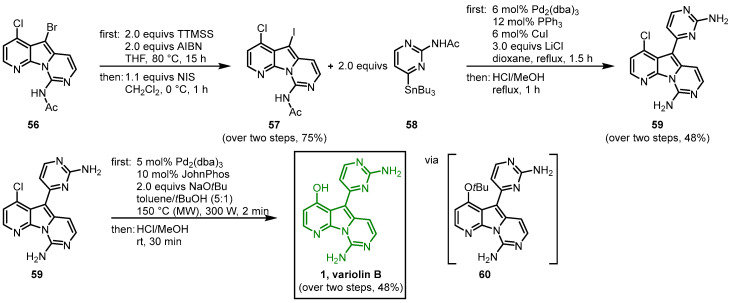
Palladium-mediated synthesis of variolin B (**1**) [51].

Next, in a debromination-iodination process, tris(trimethylsilyl)silane (TTMSS) and azobisisobutyronitrile (AIBN) and subsequently NIS were used to exchange the bromo compound **56** to the more reactive iodo derivative **57**. In a palladium-catalyzed cross-coupling reaction with the pyrimidyl stannyl reagent **58,** the C-C bond at C-5 was formed and the deprotection of both amino groups led to **59**. Then, in a palladium-promoted C-O coupling microwave (MW) reaction with sodium *tert*-butoxide, the *tert*-butyl group was introduced at C-4 to give the *tert*-butyl ether **60**, and in a final step, the *tert*-butyl moiety was cleaved to give variolin B (**1**) (Figure 12). Starting from **53,** variolin B was synthesized in seven steps with an overall yield of 5% [51,52].

### 2.2. Syntheses of Meridianins

Shortly after their discovery in 1998, it was demonstrated that meridianins exhibit strong protein kinase inhibitory properties [9,11]. Therefore, chemists quickly tried to approach the indolyl-2-aminopyridine scaffold to investigate the biological properties and for derivatization. The publication of meridianins F and G followed in 2007 [10]. Just like in the case of variolins, the biosynthetic pathway of meridianins has not been published yet.

#### 2.2.1. First Total Synthesis of Meridianins D and G by Jiang and Yang

**Figure 13:**
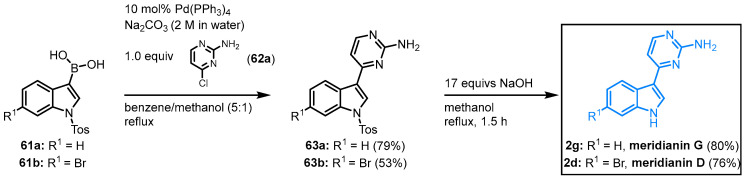
First synthesis of meridianins D and G [53].

In the early 2000s, Jiang and Yang published a straightforward synthesis of meridianins D and G. Starting from the corresponding indolyl boronic acid, **61** with 4-chloropyrimidine **62a** is the key reaction in this meridianin synthesis to furnish protected meridians **63** (Figure 13). After *N*-tosyl-deprotection of compounds **63** with sodium hydroxide, meridianin G (**2g**) is obtained in an overall yield of 63%, and meridianin D (**2d**) in an overall yield of 40% in this two-step synthesis [53]. Since meridianin G did not appear in the literature until 2004, Jiang and Yang referred to it as debromomeridianin D. 

#### 2.2.2. Synthesis of Meridianins by Fresneda and Molina

**Figure 14:**
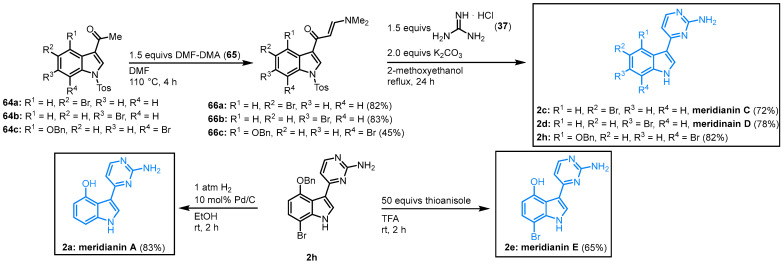
Synthesis of meridianins A, C, D and E by Molina and Fresneda [15].

Shortly after the publication of the first meridianin syntheses, Fresneda and Molina developed a facile two-step synthesis of meridianins, starting from *N*-tosyl-3-acetylindoles **64**. The reaction of **64** with dimethylformamide dimethylacetal (DMF-DMA) gave the enaminone **66**. The key step was the formation of the 2-aminopyrimidine ring by condensation of **66** with guanidine hydrochloride (**37**). Molina and Fresneda described the synthesis of meridianin D (**2d**, 65% overall yield) as well as the first total synthesis of meridianin C (**2c**, 59% overall yield) and *O*-benzyl-protected derivative **2h**. After *O*-deprotection and dehalogenation of **2h** with hydrogen and palladium on carbon, meridianin A was synthesized for the first time (**2a**, 31% overall yield) or respectively by treating **2h** with the milder deprotecting agent; no dehalogenation occurred to give the first total synthesis of meridianin E (**2e**, 24% overall yield) (Figure 14) [15,40].

To date, this method by Molina and Fresneda is the most commonly used strategy to prepare meridianin derivatives. Radwan and El-Sherbiny synthesized G amongst synthetic cyano and amidrazone analogs and investigated their cytotoxic properties [54]. Simon and Corbel used the described method to synthesize meridianins C and D together with *N*-H and *N*-alkylated analogs [55]. 

The first synthesis of meridianin F was achieved by Sperry [56] and in the same year, the synthesis of meridianin A was published by Baker alongside some analogs that were tested for CNS and antimalarial activity [57]. Bharate and Vishwakarma synthesized meridianins C and G and analogs and their antimalarial, antileishmanial, antibacterial, and antifungal activities were evaluated [12]. Hong and Lee used the protocol for the synthesis of meridianin C and derivatives substituted at the C-5 position on the indole core for the investigation of antiproliferative and pim kinase inhibitory activity [58]. A series of N-1 and C-5 functionalized derivatives, as well as meridianins C, D, and G have been synthesized by Wang, Liu, and Wang and studied for their antiviral and fungicidal properties [59], whereas Zhou and Chen synthesized meridianin C and derivatives that have been functionalized at the 2-aminopyrimidine moiety, to elucidate kinase inhibitory properties [60].

#### 2.2.3. Meridianin Synthesis by Müller via Carbonylative Alkynylation

**Figure 15:**
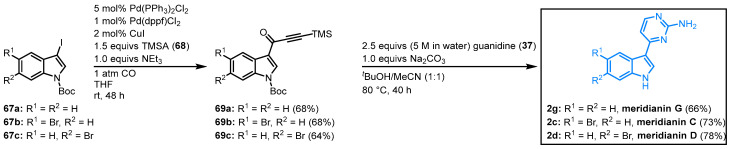
Synthesis of meridianins C, D, and G by Karpov et al. via carbonylative alkynylation [61].

In 2005, Karpov et al. published a concise synthesis of meridianins C, D, and G. *Tert*-butoxycarbonyl(Boc)-protected indoles (**67**) reacted in a palladium-catalyzed three-component carbonylative alkynylation with TMS-protected acetylene (TMSA) (**68**) to the TMS-alkynones **69**. Subsequent cyclocondensation with guanidine (**37**) concluded the meridianin synthesis as both the TMS- and the Boc-group are cleaved under the chosen reaction condition. Meridianin G was obtained with an overall yield of 45%, and meridianin C and D could be isolated with 50% overall yield (Figure 15). By choosing substituted alkynes, the C-6 of the 2-aminopyridine moiety can be easily functionalized [61]. This, and the higher yields, are favorable towards the enaminone pathway by Molina and Fresneda. 

#### 2.2.4. Meridianin Synthesis by Penoni via Indolozation of Nitrosoarenes

**Figure 16:**
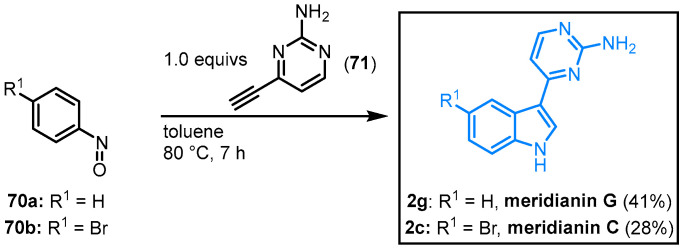
Indolization of nitrosoarenes for the synthesis of meridianins C and G by Penoni [62].

Efficiently, Penoni approached the synthesis of meridianins C and G. In a one-pot process, the corresponding nitrosobenzene **70** was reacted with 2-amino-4-ethynylpyrimidine (**71**) to give the natural products **2c** (28%) and **2g** (41%) (Figure 16) [62]. Penoni and coworkers could use their method to synthesize derivatives that are functionalized on the indole moiety as well as the pyrimidine substituent.

#### 2.2.5. Synthesis of Meridianins via One-Pot Masuda Borylation-Suzuki Coupling Sequence by Müller

**Figure 17:**
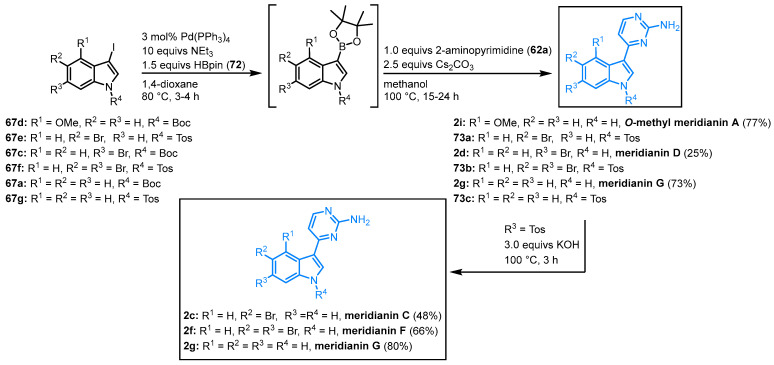
Synthesis of meridianins by Müller via MBSC sequence [63,64].

In 2011 and 2022, Müller and coworkers published a different synthetic strategy addressing meridianins. In a palladium-catalyzed Masuda borylation-Suzuki coupling (MBSC), one-pot procedure meridianins C, D, F and G, as well as the meridianin A precursor *O*-methyl meridianin A (**2i**), could be synthesized. 3-Iodo-*N*-protected indoles **67** react with pinacolyl borane (HBpin) (**72**) and without the isolation of the resulting boronic acid ester, the subsequent Suzuki coupling with 2-aminopyridine (**62a**) leads to the formation of *O*-methyl meridianin A (**2i**) and meridianins D (**2d**) and G (**2g**). The Boc-protecting group is cleaved under the Suzuki conditions. In contrast to this, *N*-tosyl-protected indoles **73** require an additional deprotecting step that can be implemented in the one-pot process. Treatment with potassium hydroxide leads to meridianins C (**2c**), F (**2f**), and G (**2g**) (Figure 17). The additional deprotecting step does not influence the overall yields. The synthesized examples have been isolated with yields ranging from 25 to 80% [63,64]. 

**Figure 18:**
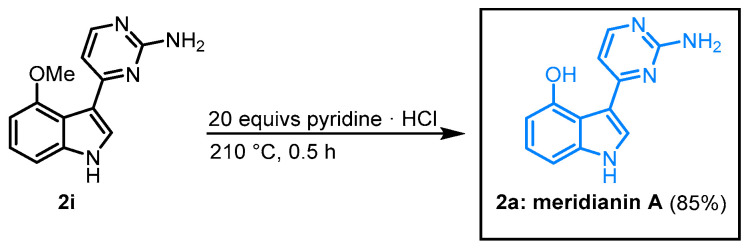
Demethylation of **2i** furnished meridianin A.

When *O*-methyl meridianin A (**2i**) was melted with pyridinium hydrochloride, the natural product **2a** could be isolated in 85% (Figure 18) [63].

#### 2.2.6. Synthesis of Meridianin F by Grainger

**Figure 19:**
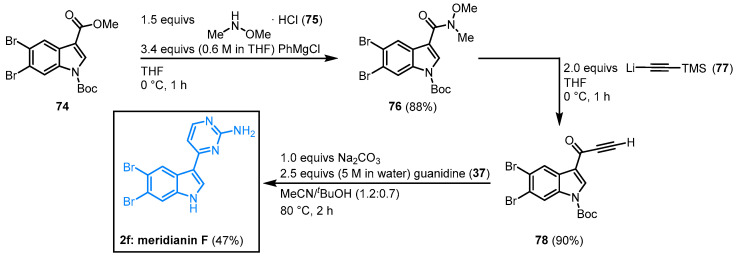
Synthesis of meridianin F by Grainger [65].

Grainger and coworkers worked on the regioselective dibromination of methyl indole-3-carboxylate and its application in the synthesis of indole building blocks. In this context, the synthesis of meridianin F was performed. Dibrominated indole **74** was reacted with *N*,*O*-dimethylhydroxylamine (**75**) to form the corresponding Weinreb amide **76**. Treatment with lithium(trimethylsilyl)acetylide (**77**) led to the formation of alkynone **78**. In the aftermath, meridianin F (**2f**) was furnished by cyclocondensation with guanidine (**37**) according to the aforementioned protocol by Müller (Section 2.2.3) in an overall yield of 37% (Figure 19) [61,65].

#### 2.2.7. Domino Amino-Palladation Reaction for the Synthesis of Meridianins C and G by Morris

**Figure 20:**
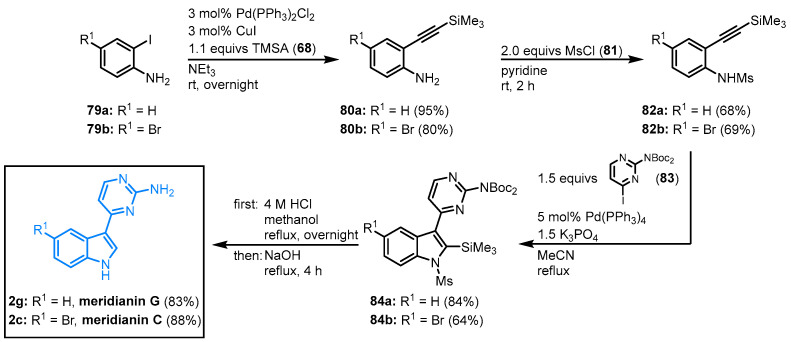
Synthesis of meridianins C and G via palladium-catalyzed domino reaction by Morris [67].

Morris and coworkers came up with a modified Cacchi protocol [66] to synthesize meridianins from readily available monocyclic precursors in a catalytic domino amino-palladation reaction [67]. The four-step synthesis starts with a Sonogashira coupling of 2-iodoaniline **79** and TMSA (**68**) to give 2-alkynyl anilines **80** followed by *N*-mesylation to give the activated sulfonamide **82**. The reaction of **82** with the *N*-Boc-protected 4-iodo-2-aminopyrimidine **83** in a Cacchi-type protocol led to the formation of the protected meridianin precursors **84**. The global deprotection was achieved in a one-pot acid/base process and furnished meridianins C (**2c**, 31% overall yield) and G (**2g**, 45% overall yield) in four steps (Figure 20). This protocol was also suitable for the generation of synthetic analogs.

### 2.3. Syntheses of Meriolins

Since variolins and meridianins not only share similar structural motifs, both natural product classes also exhibit biological activity, especially strong kinase inhibitory properties. Therefore, meriolins (**3**) have been rationally designed and can be understood as a hybrid structure of variolin B (**1**) and meridianins (**2**).

#### 2.3.1. First Synthesis of Meriolin 1 by Molina and Fresneda

**Figure 21:**
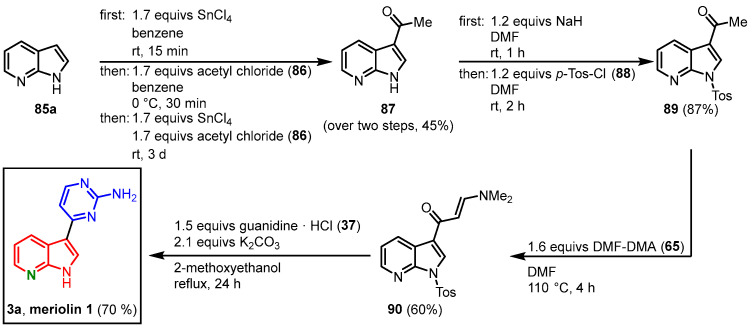
Synthesis of meriolin 1 by Molina and Fresneda [15].

With their protocol for the synthesis of meridianins in hand, Molina and Fresneda were able to extend their strategy to 7-azaindoles, leading to the first synthesis of meriolin 1 (**3a**). 7-Azaindole (**85a**) was treated with acetyl chloride (**86**) in the presence of tin (IV) tetrachloride, which afforded 3-acetyl-7-azaindole (**87**). After *N*-tosyl-protection, the enaminone **90** was furnished after the reaction of **89** with DMF-DMA (**65**) similarly to the meridianin synthesis. Cyclocondensation with guanidine (**37**) led to the 2-aminopyrimidine formation and meriolin 1 (**3a**) was obtained in the 16% overall yield (Figure 21) [15].

#### 2.3.2. Synthesis of Meriolin Derivatives by Joseph and Meijer

**Figure 22:**
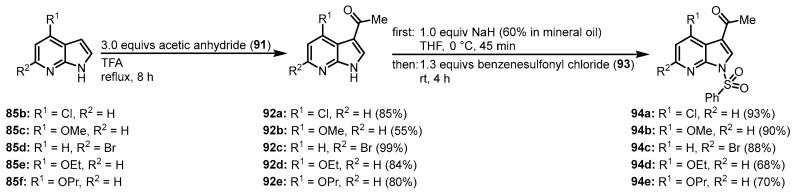
Preparation of 3-acetyl-*N*-protected intermediates **94 [14]**.

Joseph, Meijer, and coworkers used the strategy by Molina and Fresneda for the synthesis of meriolin 1 and in addition to that were able to generate a large substance library of novel meriolin derivatives. Starting from substituted 7-azaindoles **85,** acylation in 3-position was achieved by treatment with acetic anhydride (**91**) and trifluoroacetic acid. *N*-protection with benzenesulfonyl chloride (**91**) afforded the derivatives **94** (Figure 22) [14,16].

**Figure 23:**
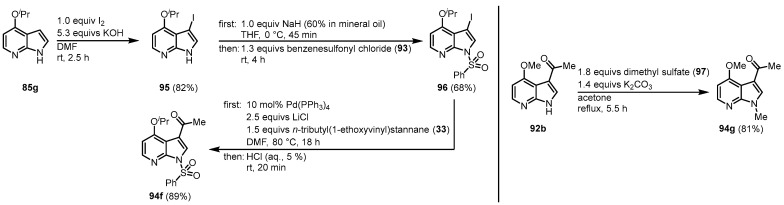
Alternative pathways to access **94f** and **94g [14]**.

In the case of **85g**, an alternative pathway was chosen to prevent *O*-demethylation under acidic conditions. After its iodination, the resulting **95** was first treated with benzenesulfonyl chloride (**93**) to give the *N*-protected intermediate **96** that was reacted with **33** in a palladium-mediated Stille cross-coupling reaction to form **94f**. Treatment of the 4-methoxy derivative **92b** with dimethyl sulfate (**97**) gave the *N*-methylated intermediate **94 g** (Figure 23).

**Figure 24:**
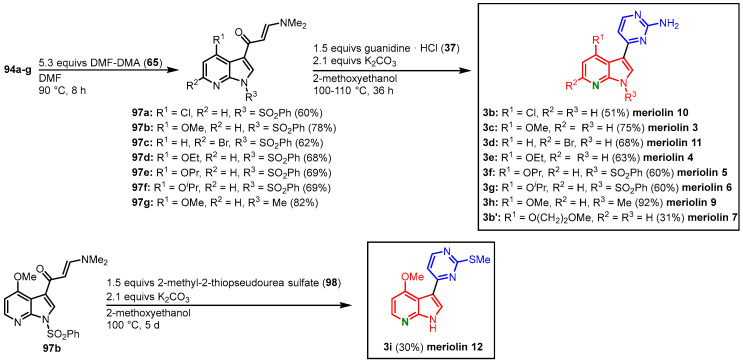
Synthesis of meriolins 3–7 and 9–12 by Joseph and Meijer [14].

The functionalized 7-azaindoles **94** were then transformed to the corresponding enaminones **97** according to the Molina and Fresneda protocol, and after cyclocondensation with guanidine (**37**), meriolins 3–7 (**3c**, **3e–g**, **3b’**) and 9–11 (**3h**, **3b**, **3d**) were obtained. Meriolin 7 (**3b’**) was isolated as a side product in the synthesis of meriolin 10 (**3b**), where a nucleophilic substitution of the chlorine substituent took place. Treatment of **97b** with 2-methyl-2-thiopseudourea sulfate (**98**) led to the formation of the 2-methylthiopyrimidine-substituted meriolin 12 (**3i**). The meriolins 3–7 and 9–12 were isolated in overall yields ranging from 12 to 37% starting from the corresponding 7-azaindole **85** (Figure 24).

**Figure 25:**
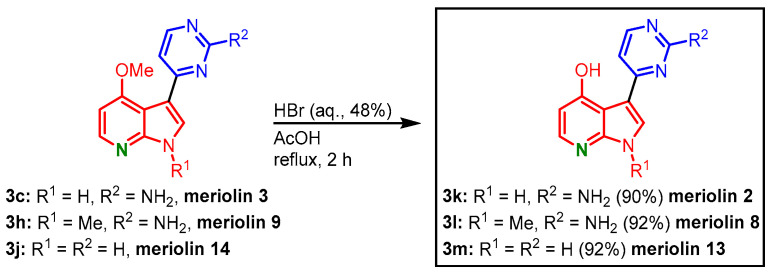
*O*-demethylation of meriolins 3, 9, and 14 gives 4-hydroxy-substituted meriolins 2, 8, and 13 [14].

The 4-methoxy-substituted meriolins **3c**, **3h**, and **3j** could be transformed to the corresponding 4-hydroxy-substituted meriolins 2 (**3k**, 26% overall yield), 8 (**3l**, 31% overall yield), and 13 (**3m**, 22% overall yield) by *O*-demethylation with hydrobromic acid in acetic acid (Figure 25) [14,16]. 

#### 2.3.3. Meriolin Syntheses by Müller via Carbonylative Alkynylation

**Figure 26:**
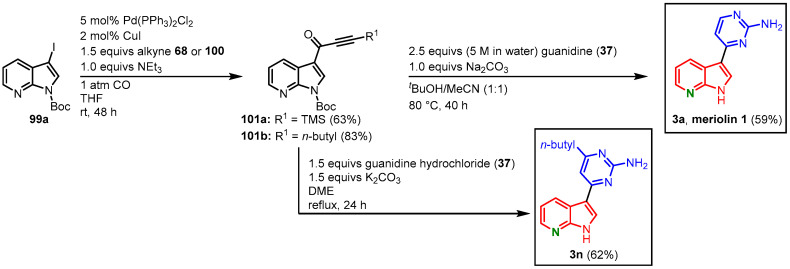
Synthesis of meriolins **3a** and **3n** via carbonylative alkynylation and subsequent pyrimidine synthesis by Müller [61,68].

The Müller approach to meridianins via carbonylative alkynylation and subsequent pyrimidine synthesis could be transferred to the synthesis of meriolins. A small library of potential kinase inhibitors has been synthesized for screenings, among them meriolin derivatives **3a** and **3n**. Therefore, 3-iodo-*N*-Boc-7-azaindole (**99a**) was transformed to the alkynones **101** in a Sonogashira coupling with TMSA (**68**) or 1-hexyne (**100**). Alkynones **101** were then cyclized with guanidine (**37**), either in a mixture of *tert*-butanol and acetonitrile or in DMF, to the meriolin derivatives **3a** (37% overall yield) and **3n** (51% overall yield) (Figure 26) [61,68].

#### 2.3.4. Three-Component Glyoxylation Decarbonylative Alkynylation Synthesis of Alkynones by Müller

**Figure 27:**
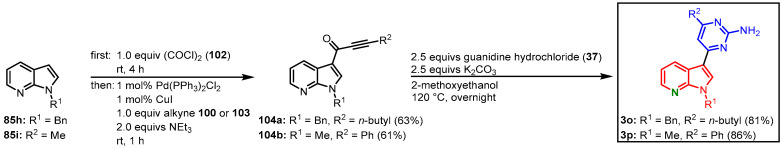
Synthesis of meriolins via three-component glyoxylation decarbonylative alkynylation by Merkul et al. [69].

Another approach by Merkul et al. to address meriolins was performed via a three-component glyoxylation alkynylation reaction, leading to *N*-benzylated and *N*-methylated meriolins **3o** and **3p**. Starting from 7-azaindoles, **85** in the first step reaction with oxalyl chloride (**102**) furnished the indole-3-glyoxyl chlorides. These reactive synthetic equivalents of acid chlorides were directly transformed to alkynones **104** in a decarbonylative Sonogashira coupling with 1-hexyne (**100**) or phenylacetylene (**103**). The cyclocondensation reaction with guanidine (**37**) went similarly as in the previously described strategy, which gave meriolins **3o** and **3p** in 51 and 52% overall yield (Figure 27) [69].

#### 2.3.5. Synthesis of Meriolins with a Suzuki Coupling as a Key Reaction by Huang

**Figure 28:**
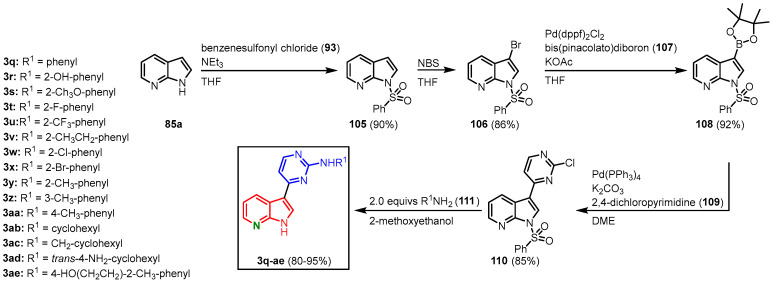
Meriolin synthesis by Huang via nucleophilic substitution on the pyrimidine moiety [70].

To investigate their kinase inhibitory effects, Huang and coworkers established a synthetic route to derivatize meriolins via a nucleophilic substitution on the pyrimidine moiety, as well as by functionalizing the N-1 and C-2 position on the azaindole moiety. 7-Azaindole (**85a**) was *N*-protected by treatment with benzenesulfonyl chloride (**93**) before it was selectively brominated in the C-3 position. Brominated and *N*-protected **106** was then transformed to the boronic acid ester **108** in a Pd(dppf)_2_Cl_2_-catalyzed Miyaura borylation with bis(pinacolato)diboron (**107**). Suzuki coupling with 2,4-dichloropyrimidine (**109**) in the presence of Pd(PPh_3_)_4_ gave **110** in superior regioselectivity. The chlorine substituent on the pyrimidine ring could then be substituted by various amines **111** in a nucleophilic aromatic substitution. Two equivalents of amine were used, as one equivalent was consumed by the concurrent cleavage of the benzenesulfonyl group. This furnished 15 meriolin derivatives (**3q-ae**) with overall yields ranging from 48 to 58% (Figure 28) [70]. 

**Figure 29:**
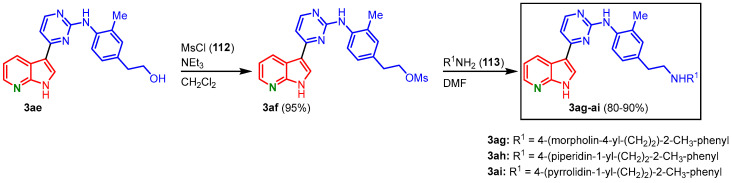
Introduction of solubilizing side chains gave the meriolin derivatives **3ag**-**ai** [70].

For the installment of solubilizing amino side chains, derivative **3ae** was treated with methanesulfonyl chloride (**112**), and the mesylate **3af** was obtained. After treatment with different amines **113,** the meriolin derivatives **3ag**-**ai** have been isolated in overall yields of 76–86% (starting from **3ae**) (Figure 29) [70].

**Figure 30:**
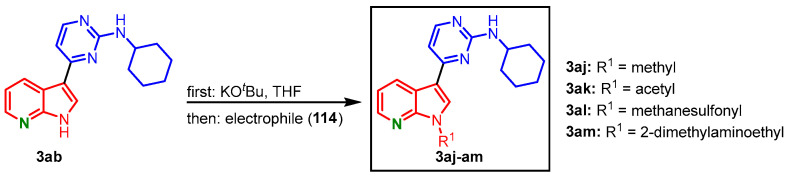
*N*-functionalization of compound **3ab** with different electrophiles gave meriolins **3aj**-**am** [70].

To assess the role of the NH group of the 7-azaindole unit in CDK1 binding, *N*-functionalization was anticipated. Compound **3ab** was treated with potassium *tert*-butoxide before it reacted with different electrophiles **114** to give the derivatives **3aj**-**am** (Figure 30) [70].

**Figure 31:**
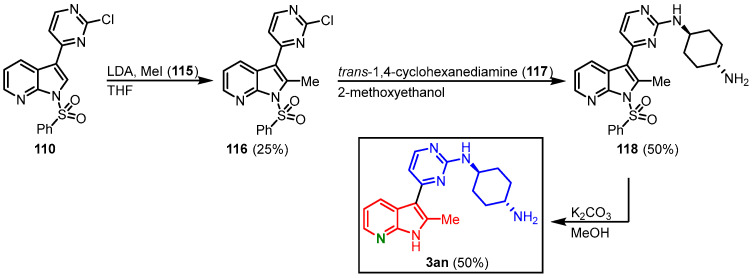
Synthesis of meriolin **3an [70]**.

Lastly, compound **110** was treated with lithiumdiisopropylamine (LDA) and methyl iodide (**115**) to introduce a methyl group in the C-2 position. After the reaction with amine **117**, an additional deprotection step was added, since the C-2 methyl group caused the *N*-benzenesulfonyl group to be stable under hot aminolysis conditions. This furnished meriolin **3an** in 6% overall yield starting from **110** (Figure 31) [70].

#### 2.3.6. Meriolin Synthesis via the Masuda borylation-Suzuki Coupling Sequence by Müller

**Figure 32:**
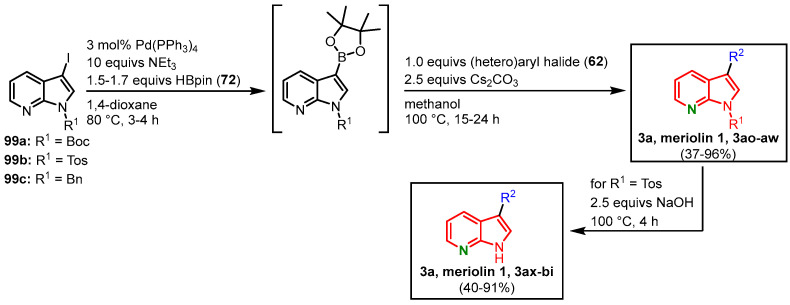
MBSC sequence for the synthesis of meriolins **3a** and **3ao**-**bi** by Müller [18,63].

The Müller group could show the versatility of the MBSC sequence by transferring their meridianin protocol to the synthesis of meriolins and other biaryl systems [18,63,64,71]. In a one-pot-process, 7-azaindoles **99** were transformed to the corresponding pinacolyl boronic acid esters in a palladium-mediated Masuda borylation. In the sense of a sequentially catalyzed reaction, a subsequent Suzuki coupling with arylhalide **62** follows (Figure 32). Under Suzuki conditions, the Boc group is concomitantly cleaved, leading to meriolin 1 (**3a**), meriolins **3ao**-**au,** and the *N*-benzylated meriolins **3av** and **3aw,** with yields ranging from 37 to 96% (Table 1). If the reaction sequence is started with *N*-tosylated azaindoles **99b** a subsequent deprotection step with a hydroxide base is required. This step can be included in the one-pot process, which led to meriolins **3a** and **3ax**-**bi** with overall yields ranging from 40 to 91% starting from 7-azaindoles **99b** (Figure 32) [18,63].

#### 2.3.7. Domino Amino-Palladation Reaction for the Synthesis of Meriolins by Morris

**Figure 33:**
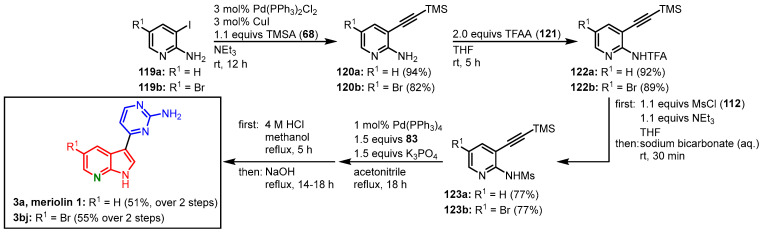
Synthesis of meriolins **3a** and **3bj** via domino amino-palladation reaction by Morris [67].

Morris and coworkers tried to adapt their meridianin protocol to the synthesis of meriolins starting from iodinated aminopyridines **119** to give 3-alkynylated 2-amino pyridines **120**. In contrast to anilines (vide supra), it was not possible to prepare the monomesylated aminopyridines directly, which required treatment with trifluoroacetyl anhydride (TFAA) (**121**) first to furnish trifluoroacetamides **122**. Reaction with mesyl chloride (**112**) led to the desired intermediate **123** that could be converted in the optimized domino reaction with *N*-Boc-4-iodopyrimidine-2-amine (**83**) and subsequent acid/base deprotection protocol to give meriolin 1 (**3a**) in 34% overall yield, as well as the 5-bromo meriolin **3bj** in 31% overall yield (Figure 33) [67].

#### 2.3.8. Metal-Free CH-Activation of a Pyrimidine and an Indolylboronic Ester by Singh

**Figure 34:**
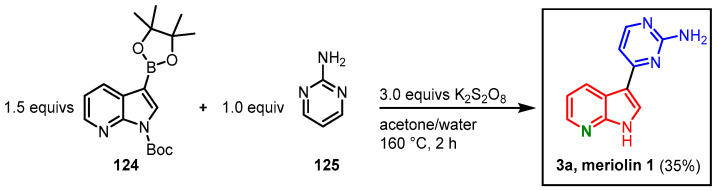
Metal-free synthesis of meriolin 1 via CH-activation by Singh [72].

In 2016, Singh presented a metal-free CH-activation approach toward the synthesis of meriolin 1. The group reported cross-coupling between diazines and related electron-deficient heteroarenes with organoboron species. Treatment of *N*-Boc-protected boronic acid ester **124** with 2-aminopyrimidine (**125**) and potassium persulfate in an acetone-water mixture led to the formation of meriolin 1 in 35% yield (Figure 34). The proposed mechanism includes the formation of a sulfate anion radical that activates the boronic acid ester and generates an azaindolyl radical. The radical reacts with the in situ-formed pyrimidinyl salt to form a radical cation. After it undergoes single electron transfer, the protonated form of the desired product is obtained [72].

#### 2.3.9. Functionalization of Meriolins via Suzuki Coupling or Nucleophilic Substitution Reactions by Singh and Malik

**Figure 35:**
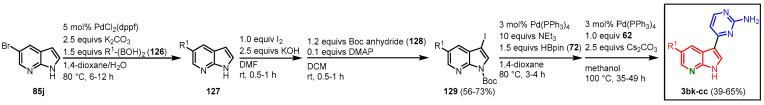
Functionalization of the C-5 position led to meriolins **3bk**-**cc [73]**.

A different approach by Singh in cooperation with Malik was more pragmatic to synthesize a large library of meriolins to establish structure-activity-relationships and determine their potency against CDKs. It was elaborated that functionalization in the C-5 position and N-1 position on the 7-azaindole unit, as well as on the pyrimidine ring, should be accomplished. Starting from 5-bromo-7-azaindole (**85j**) in a Suzuki coupling with several boronic acids, **126** led to functionalized 7-azaindoles **127** (Figure 35). After iodination and protection with Boc-anhydride (**128**) to give 3-iodo-7-azaindoles **129**, a Masuda borylation with pinacolyl borane (**72**) and subsequent Suzuki coupling with 4-chloropyrimidine-2-amine (**62**) gave the meriolin derivatives **3bk**-**cc** in 25–46% overall yield (Figure 35, Table 2) [73].

**Figure 36:**
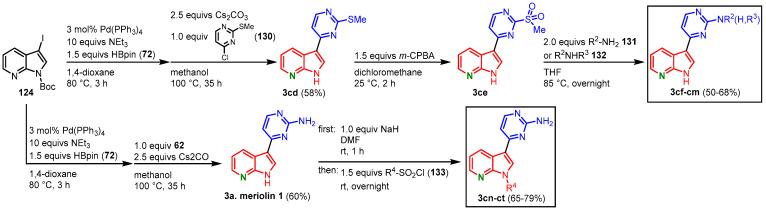
Functionalization of the pyrimidine ring and the N-1 position gave meriolins **3cf**-**ct** [73].

To derivatize the pyrimidine ring, iodinated and *N*-protected 7-azaindole **124** was transformed to the corresponding pinacolyl boronic ester and reacted with **130** in a Suzuki coupling to give compound **3cd**. The thiomethyl group was oxidized to give the sulfone **3ce** using *m*-CPBA. Nucleophilic substitution by several primary **131** or secondary amines **132** furnished meriolins **3bk**-**cc** in 29–39% overall yield (Figure 36, Table 3). To vary the substituents in the N-1 position, at first the synthesis of meriolin 1 was approached using a Masuda borylation and subsequent Suzuki coupling. After treatment with sodium hydride, reaction with different sulfonyl chlorides **133** gave meriolins **3cn**-**ct** in 39–47% overall yield (Figure 36, Table 4).

#### 2.3.10. Meriolin Synthesis via Friedel Crafts Acylation by Grädler

**Figure 37:**
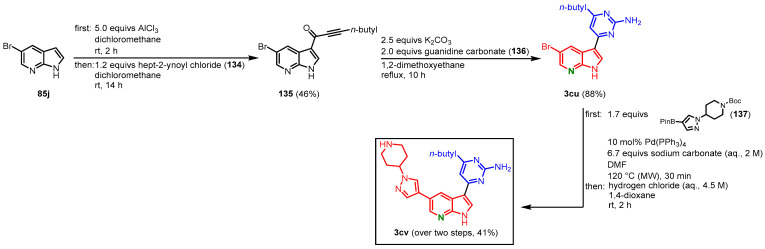
Preparation of meriolin **3cv** via Friedel Crafts acylation by Grädler [68].

Grädler and coauthors started their approach on meriolins from 5-bromo-7-azaindole (**85j**) with a Friedel Crafts acylation using aluminium chloride and acid chloride **134**. The intermediate **135** was reacted in a cyclocondensation with guanidine carbonate (**136**), which furnished meriolin **3cu** in 40% overall yield. The bromine atom in C-5 position was then employed for further derivatization. Suzuki coupling with Boc-protected pinacolyl boronic acid ester **137** and subsequent Boc-deprotection with hydrochloric acid gave meriolin **3cv** in 41% yield (Figure 37). The overall yield after three steps is 17% [68]. Using this method, as well as the carbonylative alkynylation by Müller [61], several derivatives have been synthesized and tested for their PDK1 inhibitory properties [68].

## 3. Conclusions

The naturally occurring alkaloids, variolin B (**1**) and meridianins **2**, as well as their semisynthetic hybrids meriolins **3**, represent highly active compounds with a broad spectrum of biological properties. To date, four total syntheses of variolin B (**1**), seven approaches to naturally occurring meridianins **2** and ten different strategies to meriolins **3,** are known. The described methods include cyclocondensation reactions of alkynones and enaminones with guanidine in pyrimidine syntheses and metal-free couplings, via radical reactions or the indolization of nitrosoarenes. Transition metal-catalyzed cross-coupling reactions are prominently described, among them especially Suzuki or Stille couplings. The presented strategies allow the synthesis of known compounds as well as in many cases the adaption to unknown, synthetic analogs. The natural compounds are lead structures for the synthesis of more potent synthetic analogs, such as the meriolin hybrid structures. Due to their promising and diverse biological properties, some of the described compounds are employed in pre-clinical studies, thus being potential candidates for future cancer treatments. 

## Figures and Tables

**Table 1:** Introduced heterocycles R^2^ **62** and the corresponding yields of the synthesized meriolins **3**.

Entry	Azaindole 99	R^1^	(hetero)aryl R^2^ 62	Meriolins 3 (Yield)
**1**	**99a**	H	4-pyrimidin-2-amine (**62b**)	**3a, meriolin 1** (63%)
**2**	**99a**	H	6-pyrazin-2-amine (**62c**)	**3ao** (53%)
**3**	**99a**	H	5-pyrimidin-2-amine (**62d**)	**3ap** (66%)
**4**	**99a**	H	2-pyrimidin-4-amine (**62e**)	**3aq** (37%)
**5**	**99a**	H	6-pyridin-2-amine (**62f**)	**3ar** (81%)
**6**	**99a**	H	4-pyridin-2-amine (**62g**)	**3as** (64%)
**7**	**99a**	H	2-aniline (**62h**)	**3at** (74%)
**8**	**99a**	H	4-phenol (**62i**)	**3au** (57%)
**9**	**99c**	Bn	4-pyrimidin-2-amine (**62b**)	**3av** (96%)
**10**	**99c**	Bn	4-pyridin-2-amine (**62g**)	**3aw** (93%)
**11**	**99b**	H	4-pyrimidin-2-amine (**62b**)	**3a, meriolin 1** (81%)
**12**	**99b**	H	5-pyridin-2-amine (**62j**)	**3ax** (91%)
**13**	**99b**	H	4-pyridin-2-amine (**62g**)	**3ay** (75%)
**14**	**99b**	H	*N*-benzyl-5-pyridin-2-amine (**62k**)	**3az** (77%)
**15**	**99b**	H	4-(2-methoxypyrimidine) (**62l**)	**3ba** (40%)
**16**	**99b**	H	4-pyridin-2,6-diamine (**62m**)	**3bb** (67%)
**17**	**99b**	H	5-pyrimidin-2-amine (**62d**)	**3bc** (47%)
**18**	**99b**	H	4-(2-methylthiopyrimidine) (**62n**)	**3bd** (83%)
**19**	**99b**	H	4-(6-methoxypyrimidin-2-amine) (**62o**)	**3be** (62%)
**20**	**99b**	H	4-pyrimidin-2,6-diamine (**62p**)	**3bf** (53%)
**21**	**99b**	H	*N*-benzyl-4-pyridin-2-amine (**62q**)	**3bg** (83%)
**22**	**99b**	H	2-pyrimidin-4-amine (**62e**)	**3bh** (75%)
**23 ^a^**	**99b**	H	5-isoquinolin (**62r**)	**3bi** (82%)

^a^ Suzuki coupling with DME/water.

**Table 2:** Boronic acids **126** used for the functionalization in C-5 position and the corresponding yields of meriolins **3bk**-**cc**.

Entry	Boronic Acid R^1^-B(OH)_2_ (126)	Meriolins 3 (Yield)
**1**	(4-(trifluoromethyl)phenyl)boronic acid (**126a**)	**3bk** (63%)
**2**	(4-fluorophenyl)boronic acid (**126b**)	**3bl** (65%)
**3**	(4-chlorophenyl)boronic acid (**126c**)	**3bm** (62%)
**4**	(4-(trifluoromethoxy)phenyl)boronic acid (**126d**)	**3bn** (60%)
**5**	(4-methoxyphenyl)boronic acid (**126e**)	**3bo** (59%)
**6**	(4-(methylthio)phenyl)boronic acid (**126f**)	**3bp** (54%)
**7**	(3-fluorophenyl)boronic acid (**126g**)	**3bq** (55%)
**8**	*m*-tolylboronic acid (**126h**)	**3br** (50%)
**9**	(3-(trifluoromethyl)phenyl)boronic acid (**126i**)	**3bs** (58%)
**10**	(2-(methylthio)phenyl)boronic acid (**126j**)	**3bt** (59%)
**11**	(2-ethylphenyl)boronic acid (**126k**)	**3bu** (48%)
**12**	naphthalen-1-ylboronic acid (**126l**)	**3bv** (60%)
**13**	(2-methoxynaphthalen-1-yl)boronic acid (**126m**)	**3bw** (55%)
**14**	furan-3-ylboronic acid (**126n**)	**3bx** (48%)
**15**	thiophen-3-ylboronic acid (**126o**)	**3by** (44%)
**16**	pyridin-3-ylboronic acid (**126p**)	**3bz** (45%)
**17**	benzo[*b*]thiophen-2-ylboronic acid (**126q**)	**3ca** (40%)
**18**	benzofuran-2-ylboronic acid (**126r**)	**3cb** (39%)
**19**	(5-methoxy-1*H*-indol-2-yl)boronic acid (**126s**)	**3cc** (45%)

**Table 3:** Primary **131** and secondary amines **132** used for the functionalization of the pyrimidine ring and the corresponding yields of meriolins **3cf**-**cm**.

Entry	Amine R^2^-NH_2_ (131) or R^2^NHR^3^ (132)	Meriolins 3 (Yield)
**1**	2-phenylethan-1-amine (**131a**)	**3cf** (65%)
**2**	2-(4-methoxyphenyl)ethan-1-amine (**131b**)	**3cg** (60%)
**3**	2-(3,4-dimethoxyphenyl)ethan-1-amine (**131c**)	**3ch** (68%)
**4**	2-(1*H*-indol-3-yl)ethan-1-amine (**131d**)	**3ci** (50%)
**5**	pyrrolidine (**132a**)	**3cj** (52%)
**6**	piperidine (**132b**)	**3ck** (52%)
**7**	morpholine (**132c**)	**3cl** (60%)
**8**	1-methylpiperazine (**132d**)	**3cm** (54%)

**Table 4:** Sulfonyl chlorides **133** for the functionalization in N-1 position and the corresponding yields of meriolins **3cn**-**ct**.

Entry	Sulfonyl Chloride R^4^-SO_2_Cl (133)	Meriolins 3 (Yield)
**1**	4-fluorobenzenesulfonyl chloride (**133a**)	**3cn** (70%)
**2**	4-bromobenzenesulfonyl chloride (**133b**)	**3co** (70%)
**3**	4-(trifluoromethyl)benzenesulfonyl chloride (**133c**)	**3cp** (77%)
**4**	4-(trifluoromethoxy)benzenesulfonyl chloride (**133d**)	**3cq** (80%)
**5**	4-acetamidobenzenesulfonyl chloride (**133e**)	**3cr** (71%)
**6**	2,3-dihydrobenzo[*b*][1,4]dioxine-6-sulfonyl chloride (**133f**)	**3cs** (79%)
**7**	1-methyl-1*H*-imidazole-5-sulfonyl chloride (**133g**)	**3ct** (65%)

## Data Availability

Not applicable.
